# Rates of mental health concerns among individuals assessed at the GoodHope Ehlers-Danlos Syndrome Clinic

**DOI:** 10.1186/s13023-025-03550-5

**Published:** 2025-02-14

**Authors:** P. Maxwell Slepian, Kristina Axenova, Molly McCarthy, Rachel Siegal, Keisha Gobin, Aliza Weinrib, Stephanie Buryk-Iggers, Daniel Santa Mina, Laura McGillis, Nimish Mittal, Joel Katz, Hance Clarke

**Affiliations:** 1 GoodHope Ehlers-Danlos Syndrome Clinic, Toronto General Hospital, 11 PMB 100D, 200 Elizabeth St, Toronto, ON, CA M5G 2C4 Canada; 2 Department of Anesthesia and Pain Management, Toronto General Hospital, Toronto, Ontario Canada; 3 Department of Anesthesiology and Pain Medicine, University of Toronto, Toronto, Ontario Canada; 4 Krembil Research Institute, University Health Network, Toronto, Ontario Canada; 5 Department of Psychology, York University, Toronto, Ontario Canada; 6 St. Joseph’s Healthcare, Hamilton, Canada; 7 Faculty of Kinesiology and Physical Education, University of Toronto, Toronto, Canada

**Keywords:** Ehlers-danlos syndrome, Joint hypermobility, Hypermobility spectrum disorder, Psychology, Anxiety

## Abstract

Past research has indicated that individuals with Ehlers-Danlos Syndromes (EDS) and Generalized Hypermobililty Spectrum Disorder (G-HSD) report psychological and psychiatric symptoms, particularly anxiety disorders and depressive symptoms, at much greater rates than the general population. However, these studies have been primarily conducted in small samples at European centres. We report a retrospective chart review from 1035 consecutive patients (88% female) assessed for EDS/G-HSD at the GoodHope EDS Clinic at Toronto General Hospital between June 2019 and June 2021. Prior to assessment, all patients completed self-reported mental health screening questions, the Inventory of Depressive and Anxiety Symptoms – Dysphoria scale, and the Borderline Symptom List-23. The majority of patients reported current or past anxiety or depressive symptoms (53–87%), and a substantial minority reported significant mental health concerns, including Posttraumatic Stress Disorder (4.7–34.8%), disordered eating (19%), self-harm (3-29.2%), and suicidal behaviour (7.8–18.6%). Patients did not differ by diagnostic category on self-report measures of dysphoria or borderline symptoms. Individuals with G-HSD reported higher rates of anxiety and depression in clinical interview than those diagnosed with non-hypermobile EDS, and endorsed a higher rate of having “struggled with anxiety or depression” on the mental health screening questionnaire than individuals not diagnosed with EDS/G-HSD. No other differences emerged across diagnostic groups. These findings highlight the need for psychological support for individuals with EDS or G-HSD.

## Introduction

Ehlers-Danlos Syndromes (EDS) and Generalized Hypermobility Spectrum Disorder (G-HSD) are a group of hereditary connective tissue disorders that are typically associated with a range of physiological features including unstable joints that easily sublux/dislocate, stretchy/fragile skin, and organ/systemic dysfunction [1]. These features are associated with myriad symptoms and co-morbid conditions, including chronic pain and recurrent acute pain, gastrointestinal distress, dysautonomia, and respiratory symptoms [1]. Among these common co-morbidities, a number of psychological disorders have also been noted to commonly co-occur with EDS/G-HSD [2–4]. 

Recognition that psychological symptoms are an important aspect of EDS/G-HSD emerged out of clinical observation [2]. Initial reports focused primarily on anxiety symptoms in individuals diagnosed with Joint Hypermobility Syndrome (JHS), based on the Beighton Criteria [5, 6]. These studies identified higher rates of panic disorder, agoraphobia, and simple phobias in individuals with JHS compared to controls recruited from a rheumatology clinic [5, 6]. Further case-controlled studies have confirmed an association between hypermobility and anxiety disorders, and have also linked hypermobility to an increased risk of major depression [7]. Case reports have also linked EDS/G-HSD with disordered eating; and studies of psychiatric populations have identified greater hypermobility among individuals diagnosed with anxiety, ADHD, or psychosis compared to controls [3]. 

However, there are notable limitations in these studies. Given the rapidly changing definitions and diagnostic criteria for EDS and hypermobility [1], the majority of these studies were conducted using dated diagnostic criteria, particularly for JHS, which is no longer recognized by the International EDS Consortium as a clinical diagnosis [7]. Findings from these studies continue to be generalized to EDS/G-HSD without re-evaluation in the context of the 2017 diagnostic criteria. Moreover, the case-control approach taken in the majority of these studies rarely reflects the medical complexity typically seen in EDS/G-HSD. That is, comparison groups do not control for symptoms such as pain and dysautonomia that are also linked to psychological distress [5, 6]. 

Recently, the GoodHope Ehlers-Danlos Syndrome Clinic was established at Toronto General Hospital [8]. This multi-disciplinary program was established for the purpose of timely diagnosis and management to improve lives of people living with EDS and G-HSD. Patients suspected to have EDS or hypermobility are referred by family physicians or other specialists. Diagnostic experts conduct detailed clinical assessment, physical examination and order genetic testing if necessary. Patients who are diagnosed with any subtype of EDS or G-HSD are then advised a comprehensive treatment plan including visits to subspecialty EDS physicians (pain medicine, gastroenterology, respirology, cardiology, allergy and immunology) and professionally-guided self-management programs (psychology, physiotherapy and rehabilitation, dietitian, and social work). As part of the program intake, all patients are asked about mental health history and complete several validated self-report measures focused on mental health. The current study is a retrospective analysis of individuals assessed at the GoodHope EDS Clinic and aims to describe rates of mental health concerns and identify whether mental health concerns differ by diagnostic group; non-hypermobile EDS, hEDS, G-HSD, or not diagnosed with EDS/G-HSD (i.e. no signs of hypermobility or collagen dysfunction).

## Methods

### Participants

Participants include all consecutive patients assessed at the GoodHope Ehlers-Danlos Syndrome (EDS) Clinic between June 2018 and June 2021 as part of a retrospective chart review. A total of 1035 patients (88% female) were assessed at the GoodHope EDS clinic over the study period outlined, making them eligible for inclusion.

### Procedure

Patients from across Ontario were referred to the GoodHope EDS clinic by family physicians or other specialists for assessment of suspected EDS or G-HSD. As part of the intake process, patients completed self-report measures of mental health prior to their initial visit to the clinic. During their initial assessment, the physician or nurse practitioner took a detailed medical and social history, including questions on mental health history. A physical examination focused on identifying markers of hypermobility and connective tissue dysfunction was then performed. If necessary, patients were referred for genetic testing at this time. Following this assessment, (and genetic testing where applicable) patients were diagnosed with EDS, G-HSD or were found not to meet the 2017 EDS criteria and discharged from the program.

### Measures

Mental health history was assessed using two methods. The first was an in-house, self-report screening measure that asked participants to respond (Yes/No) to the following questions: (1) “Have you ever struggled with anxiety or depression”, (2) “Have you ever taken medication for anxiety/depression”, (3) “Have you ever had an eating disorder”, (4) “Do you now or have you in the past suffered from Post-Traumatic Stress Disorder”, (5) “Have you ever hurt yourself on purpose”, (6) “Have you ever tried to end your life”, (7) “Have you ever spent time in the hospital for psychiatric care”, and 7) “Have you ever had treatment for mental health in the past?”. Second, patients were asked in a clinical interview if they had a history (Yes/No) of anxiety, depression, Post-Traumatic Stress Disorder, Obsessive-Compulsive Disorder, or a personality disorder.

Dysphoria was assessed using the Inventory of Depressive and Anxiety Symptoms – Dysphoria subscale (IDAS-D) [9]. The IDAS-D is 10-item a scale demonstrating a strong ability to screen for the presence of internalizing psychopathology [10] – specifically relevant to general distress (i.e., dysphoria). Empirically-established cut-offs on the IDAS-D have been identified to screen for likely internalizing disorder, and a cut-off score of 28.5 was determined to maximize diagnostic specificity. Moreover, the IDAS-D is a more sensitive and specific screening measure than commonly-used scales such as the Beck Depressive Inventory – II and the Beck Anxiety Inventory [10]. 

Emotion dysregulation was assessed using the Borderline Symptom List (BSL-23) [11]. The BSL-23 is a well-established self-report instrument based on the criteria of the Diagnostic and Statistical Manual of Mental Disorders 5th Edition for the revised diagnostic criteria for borderline personality disorder (BPD), the experiences of both clinical experts, and BPD patients. This instrument is used to assess the severity of 23 feelings and experiences typically reported by BPD patients [11]. Items are averaged to create a final score with higher score indicative of higher levels of borderline psychopathology. In the current sample, the alpha coefficient value is 0.95 for the BSL-23 – reflecting excellent internal consistency. A clinical cut-off score of 1.5 has been recommended to discriminate between individuals with BPD and other clinical populations [12].

Pain, fatigue, and physical function were assessed using the Multidimensional Health Assessment Questionnaire (MDHAQ) [13]. The MDHAQ includes single numerical ratings scales measuring pain due to illness in the past 2 weeks, from 0, *no pain*, to 10, *pain as bad as it could be*, and a single item measuring fatigue over the past week, from 0, *fatigue is not a problem*, to 10, *fatigue is a major problem*. The MDHAQ also includes 10 items assessing physical function (e.g. “Get in and out of bed”) on a 4-point Likert scale, from 0, *without any difficulty*, to 3, *unable to do*. Items are summed and then divided by 3 to create a final score that ranges from zero to 10, with higher scores indicating poorer physical function [13]. The physical function subscale demonstrated excellent internal consistency in the current sample, α = 0.90.

The Composite Autonomic Symptom Score-31 (COMPASS 31) was used to assess self-reported autonomic dysfunction. The COMPASS-31 is a 31-item questionnaire consisting of six domains: orthostatic intolerance (4 items), vasomotor dysfunction (3 items), secretomotor dysfunction (4 items), gastrointestinal system dysfunction (12 items), bladder dysfunction (3 items), and pupillomotor dysfunction (5 items) [14]. Patients are asked to rate the presence, frequency, and severity of symptoms. The total score ranges from 0 to 100, with higher scores indicating greater autonomic dysfunction.

### Data analysis

For the purposes of the current study, physician/NP diagnosis was simplified into four diagnoses, hEDS, G-HSD, non-hypermobile EDS subtypes diagnoses, not-EDS/G-HSD (i.e. not diagnosed with EDS or G-HSD). Non-hypermobile EDS subtypes were combined into a single diagnostic group to allow for quantitative analysis of mental health concerns and, unlike hEDS which can be diagnosed only by clinical assessment, all of these individual syndromes have a known genetic mechanism of collagen dysfunction. These subtypes were combined in analyses as previous has indicated that individuals with genetically-confirmed EDS experience higher quality of life and lower mental health symptoms than individuals with hEDS [15]. Chi -square tests were used to examine differences in binary mental health variables by diagnostic category. Pairwise chi-squared tests were used to follow-up significant omnibus tests. Within each group of follow-up tests, a Bonferroni correction was used to control family-wise Type I error rate (i.e., 0.05/number of tests). A series of one-way analyses of variance (ANOVAs) was used to examine differences in symptom severity between non-hypermobile EDS, hEDS, G-HSD, and not-EDS/G-HSD.

## Results

### Sample characteristics

A summary of sample characteristics is presented in Table 1. Of the 1035 consecutive cases reviewed, 42 (4.1%) were diagnosed with a non-hypermobile EDS (Classical EDS, *n* = 18; Classical-like EDS, *n* = 2; Vascular EDS, *n* = 16; Arthrochalasia EDS, *n* = 1; Kyphoscoliotic EDS, *n* = 2; Musculocontractural EDS, *n* = 1; Periodontal EDS, *n* = 2), 81 (7.8%) were diagnosed with hEDS, 410 (39.6%) were diagnosed with G-HSD, and 502 (48.5%) were not to meet diagnostic criteria for EDS or G-HSD (i.e. not-EDS/G-HSD). Across diagnostic categories, the average age was 35.4 years (SD = 11.9), and the majority were female sex (88.5%). Education, employment, and marital status are described in Table 1. Pairwise comparisons indicated that individuals with non-hypermobile EDS subtypes and those with not-EDS/G-HSD were older at the time of their initial EDS clinic visit than those with hEDS or G-HSD, all *p*’s < 0.008. There was a greater proportion of males among individuals with non-hypermobile EDS than any other diagnostic category, all *p’s* < 0.001. Pain intensity (partial η^2^ = 0.02), fatigue (partial η^2^ = 0.02), and autonomic dysfunction (partial η^2^ = 0.04) differed by diagnostic category, all *p*’s < 0.05. Follow-up pairwise tests with Bonferroni correction indicated that individuals with hEDS, G-HSD, and not-EDS/G-HSD reported greater pain, fatigue, and autonomic dysfunction than individuals with non-hypermobile EDS, all *p*’s < 0.008.

**Table 1:** Sample characteristics

	Total Sample (*n* = 1035)	Non-hypermobile EDS (*n* = 42)	hEDS (*n* = 81)	G-HSD (*n* = 410)	Not diagnosed EDS/G-HSD (*n* = 502)	*P*-value
Sex, *n* (%)						
Female Male	916 (88.5) 119 (11.5)	27 (64.3) 15 (35.7)	75 (92.6) 6 (7.4)	372 (90.7) 38 (9.3)	442 (88) 60 (12)	<0.001**
Age, Mean, SD	35.3 (12)	39.6 (13.9)	33.02 (10.5)	33.16 (11.1)	36.96 (12.3)	< 0.001**
Education Level, *n* (%)^a^						
High School College/University Post-Graduate	195 (22) 551(62.2) 140 (15.8)	11 (32.4) 19 (55.9) 4 (11.8)	19 (26) 38 (52.1) 16 (21.9)	88 (24.4) 214 (59.3) 59 (16.3)	77(18.4) 280 (70) 61 (14.6)	0.06
Employment Status, *n* (%)^b^						
Disability/Benefits Employed Homemaker Looking for work Student	259 (26.8) 439 (45.4) 66 (6.8) 47 (4.9%) 156 (16.1)	10 (26.3) 23 (60.5) 1 (2.6) 1 (2.6) 3 (7.9)	20 (25.3) 39 (49.4) 3 (3.8) 5 (6.3) 12 (15.2)	97 (24.5) 176 (44.4) 26 (6.6) 24 (6.1) 73 (18.4)	132 (29.1) 201 (44.3) 36 (7.9) 17 (3.7) 68 (15)	0.34
Marital Status, *n* (%)^c^						
Common law Divorced Married Separated Single Widowed	108 (11.3) 33 (3.5) 323 (33.8) 25 (2.6) 462 (45.3) 5 (0.5)	3 (8.3) 2 (5.6) 19 (52.8) 1 (2.8) 11 (30.6) 0 (0)	13 (16.9) 3 (3.9) 19 (24.7) 1 (1.3) 41 (53.2) 0 (0)	40 (10.4) 15 (3.9) 118 (30.7) 8 (2.1) 202 (52.6) 1 (0.3)	52 (11.3) 13 (2.8) 167 (36.4) 15 (3.3) 208 (45.3) 4 (0.9)	0.15
Beighton Score, Mean (SD)	3.9 (2.2)	3.5 (2.6)	6.1 (1.7)	5.2 (1.4)	2.4 (1.6)	< 0.001**
Pain^d^ Fatigue^d^ Physical Function^d^	5.9 (2.4) 7 (2.6) 2.7 (1.8)	4.5 (3.1) 5.2 (3.4) 2.1 (2.0)	6.3 (2.0) 7.0 (2.6) 2.9 (1.9)	6.0 (2.1) 7.3 (2.4) 2.7 (1.8)	5.9 (2.6) 6.9 (2.7) 2.7 (1.8)	< 0.001** 0.001** 0.157
Autonomic Dysfunction^e^, Mean (SD)	26.7 (9.3)	18.6 (12.6)	26.9 (7.7)	28.0 (8.7)	26.61 (9.4)	0.002**

Notes. *p < 0.0.05, **p < 0.01, ***p < 0.001; ^a^Missing *n* = 149; ^b^Missing *n* = 68; ^c^Missing *n* = 79 ^d^From Multidimensional Health Assessment Questionnaire Total *n* = 950, non-hypermobile EDS *n* = 37, hEDS *n* = 74, G-HSD *n* = 384, Not diagnosed EDS/G-HSD *n* = 455; ^e^Autonomic Dysfunction Total *n* = 401, non-hypermobile EDS *n* = 14, hEDS *n* = 31, G-HSD *n* = 166, Not diagnosed EDS/G-HSD *n* = 190

### Mental health screening questionnaire

Table 2 describes rates of mental health concerns for the overall sample and by diagnostic category. Across groups, there was a high prevalence of depression/anxiety (87.5%), disordered, eating (19%), PTSD (34.8%), self-harm (29.2%), and suicidal behavior (18.6%). Patients differed by diagnostic category in rate of endorsing “have you ever struggled with anxiety or depression”, Χ^2^(3, *n* = 781) = 15.03, *p* = 0.002, “have you ever taken medication for anxiety or depression”, Χ^2^(3, *n* = 781) = 8.08, *p* = 0.04, “have you ever hurt yourself on purpose”, Χ^2^(3, *n* = 781) = 8.87, *p* = 0.03. Follow-up pairwise tests indicate that individuals diagnosed with G-HSD had greater odds of having “struggled with anxiety or depression” than those not diagnosed with EDS/G-HSD, OR = 2.39, Χ^2^(1, *n* = 688) = 12.35 *p* < 0.001, or those with non-hypermobile EDS subtypes, OR = 3.72, Χ^2^(1, *n* = 361) = 7.49, *p =* 0.006. No other pairwise comparisons were significant with Bonferroni correction.

**Table 2:** Rates of mental health concerns reported on mental health screening questionnaire

	Total Sample (*n* = 781)	Non-hypermobile EDS (*n* = 26)	hEDS (*n* = 67)	G-HSD (*n* = 335)	Not diagnosed EDS/G-HSD (*n* = 353)	*P*-value
1) Have you ever struggled with anxiety or depression?						
Yes No	683 (87.5) 98 (12.5)	20 (47.6) 6 (23.1)	57 (85.1) 10 (14.9)	310 (92.5) 25 (7.5)	296 (83.9) 57 (16.1)	0.002**
2) Have you ever taken medication for anxiety or depression?						
Yes No	553 (70.8) 228 (29.2)	14 (53.8) 12 (46.2)	45 (67.2) 22 (32.8)	252 (75.2) 83 (24.8)	242 (68.6) 111 (31.4)	0.044*
3) Have you ever had an eating disorder?						
Yes No	148 (19) 633 (81)	3 (11.5) 23 (88.5)	17 (25.4) 50 (74.6)	72 (21.5) 263 (78.5)	56 (15.9) 297 (84.1)	0.097
4) Do you now or have you in the past suffered from Post-Traumatic Stress Disorder?						
Yes No	272 (34.8) 509 (65.2)	6 (23.1) 20 (76.9)	21 (31.3) 46 (68.7)	126 (37.6) 209 (62.4)	119 (33.7) 234 (66.3)	0.351
5) Have you ever hurt yourself on purpose?						
Yes No	228 (29.2) 553 (70.8)	4 (15.4) 22 (84.6)	23 (34.3) 44 (65.7)	112 (33.4) 223 (66.6)	89 (25.2) 264 (74.8)	0.031*
6) Have you ever tried to end your life?						
Yes No	145 (18.6) 636 (81.4)	3 (11.5) 23 (88.5)	15 (22.4) 52 (77.6)	62 (18.5) 273 (81.5)	65 (18.4) 288 (81.6)	0.682
7) Have you ever spent time in the hospital for psychiatric care?						
Yes No	144 (18.4) 637 (81.6)	3 (11.5) 23 (88.5)	14 (20.9) 53 (79.1)	65 (19.4) 270 (80.6)	62 (17.6) 291 (82.4)	0.687
8) Have you ever had treatment for mental health in the past?						
Yes No	470 (60.2) 311 (39.8)	13 (50.0) 13 (50.0)	38 (56.7) 29 (43.3)	209 (62.4) 126 (37.6)	210 (59.5) 142 (40.5)	0.530

Note. *p < 0.0.05, **p < 0.01, ***p < 0.001

### Clinician history

Table 3 describes rates of mental health diagnoses reported during clinician history taking for the total sample and by diagnostic category. Across diagnostic groups, the majority of patients reported anxiety (62%) and depression (53%), whereas a smaller proportion of patients reported PTSD (4.7%), personality disorders (1.5%), obsessive-compulsive disorder (2.1%), history of suicide attempts (1.9%), current suicidal ideation (7.8%), and self-harm (3%). Patient reported history of anxiety, Χ^2^(3, *n* = 1035) = 4.36, *p* = 0.002, and depression, Χ^2^(3, *n* = 1035) = 10.16, *p* = 0.017, differed by diagnostic category. Follow-up pairwise tests indicated that individuals diagnosed with G-HSD had greater odds of reporting a past diagnosis of depression, OR = 2.77, Χ^2^(1, *n* = 448) = 8.91, *p =* 0.003, or anxiety, OR = 3.07, Χ^2^(1, *n* = 448) = 11.77, *p =* 0.001, than individuals with non-hypermobile EDS subtypes. No other pairwise comparisons were significant with Bonferroni correction. There were no differences by diagnostic category in patient reported history of PTSD, personality disorder, Obsessive Compulsive Disorder, all *p*’s > 0.05.

**Table 3:** Rates of mental health diagnoses from clinician history note

	Total Sample (*n* = 1020)	Non-hypermobile EDS (*n* = 40)	hEDS (*n* = 81)	G-HSD (*n* = 408)	Not diagnosed EDS/G-HSD (*n* = 491)	*P*-Value
Anxiety						
Yes No	632 (62.0) 388 (38.0)	16 (40.0) 24 (60.0)	46 (56.8) 35 (43.2)	274 (66.8) 134 (32.7)	296 (60.3) 195 (39.7)	0.002**
Depression						
Yes No	541 (53.0) 479 (47.0)	13 (32.5) 27 (67.5)	40 (49.4) 41 (50.6)	233 (57.1) 175 (42.9)	255 (51.9) 236 (48.1)	0.017*
PTSD						
Yes No	48 (4.7) 972 (95.3)	0 (0) 40 (100.0)	3 (3.7) 78 (96.3)	23 (5.6) 385 (94.4)	22 (4.5) 469 (95.5)	0.391
Personality Disorder						
Yes No	15 (1.5) 1005 (98.5)	0 (0) 40 (100.0)	0 (0) 81 (100.0)	8 (2.0) 400 (98.0)	7 (1.4) 484 (98.6)	0.477
Obsessive Compulsive Disorder						
Yes No	21 (2.1) 999 (97.9)	0 (0) 40 (100.0)	0 (0) 81 (100.0)	9 (2.2) 399 (97.8)	12 (2.4) 479 (97.6)	0.400
Past Suicide Attempts						
Yes No	19 (1.9) 1001 (98.1)	0 (0) 40 (100.0)	1 (1.2) 80 (98.8)	12 (2.9) 396 (97.1)	6 (1.2) 485 (98.8)	0.201
Current Suicidal Thoughts/Ideation						
Yes No	80 (7.8) 940 (92.2)	2 (5.0) 38 (95.0)	9 (11.1) 72 (88.9)	29 (7.1) 379 (92.9)	40 (8.1) 451 (91.9)	0.570
History/Current Self-Harm						
Yes No	31 (3.0) 989 (97.0)	1 (2.5) 39 (97.5)	2 (2.5) 79 (97.5)	15 (3.7) 393 (96.3)	13 (2.6) 478 (97.4)	0.814

*p < 0.0.05, **p < 0.01, ***p < 0.001

### Dysphoria

Patient scores on the IDAS-D did not differ by diagnostic category, *F*(3,741) = 1.05, *p* = 3.68. Self-reported dysphoria was also evaluated using a diagnostic cut off for likely internalizing disorder (IDAS-D > 28.5). Across groups, a large minority of patients (43.4%) screened positive for likely internalizing disorder. There were no differences in rates of likely internalizing disorder among individuals with hEDS (36%), non-hypermobile EDS (40.9%), G-HSD (44.4%), or those not diagnosed with EDS/G-HSD (43.4%), Χ^2^(3, *n* = 745) = 0.85, *p* = 0.84. Scores on the IDAS-D are summarized in Table 4.

**Table 4:** Dysphoria and Borderline symptoms by diagnosis

	Total Sample	Non-hypermobile EDS	hEDS	G-HSD	Not diagnosed EDS/G-HSD	*P*-value
**Dysphoria** ^**a**^ Total Score, Mean (SD)						
% above Clinical Cutoff (N)	27.0 (9.7) 43.4 (323)	23.8 (10.3) 36 (9)	26.4 (9.2) 40.9 (27)	27.2 (9.5) 44.4 (142)	27.1 (10.0) 43.4 (145)	0.57 0.84
**Borderline Symptoms** ^**b**^						
Mean Score (SD) % above Clinical Cutoff (N)	0.88 (0.8) 20.1 (151)	0.52 (0.5) 7.7 (2)	0.89 (0.7) 16.9 (11)	0.91 (0.8) 22.5 (73)	0.88 (0.75) 19.5 (65)	0.09 0.25

Notes. *p < 0.0.05, **p < 0.01, ***p < 0.001; ^a^Dysphoria Total *n* = 745, non-hypermobile EDS *n* = 25, hEDS *n* = 66, G-HSD *n* = 320, Not diagnosed EDS/G-HSD *n* = 334; ^b^Borderline Symptoms Total *n* = 750, non-hypermobile EDS *n* = 26, hEDS *n* = 65, G-HSD *n* = 325, Not diagnosed EDS/G-HSD *n* = 334

### Borderline symptoms

Patient reported borderline symptoms did not differ by diagnostic category, *F*(3,749) = 2.18, *p* = 0.09. Borderline symptoms were also evaluated using a diagnostic cut-off score (1.5), that has been found to distinguish individuals with BPD from other clinical populations. Approximately one-fifth of the sample (20.1%) screened positive for likely BPD. There were no differences in rates of likely borderline personality disorder among individuals with hEDS (16.9%), non-hypermobile EDS subtypes (7.7%), G-HSD (22.5%), or not-hEDS/GHSD (19.5%), Χ^2^(3, *n* = 750) = 4.1, *p* = 0.25. Scores on the BSL-23 are summarized in Table 4.

## Discussion

The current study presents a retrospective review of mental health concerns among patients assessed at the GoodHope Ehlers-Danlos Syndrome Program between July, 2019 and June, 2021. As part of an assessment for EDS and hypermobility, patients were asked to self-report on mental health history and complete measures of dysphoria and borderline personality disorder symptoms. Patients universally reported extremely high rates of mental health concerns. 87.5% reported a history of anxiety or depression. Moreover, the report of a history of PTSD, disordered eating, self-harm, and current or past suicidal ideation, and history of suicide attempts were all well above lifetime prevalence of these disorders in the general population [16]. Likewise, on symptom self-report (IDAS-D, BSL-23), a large minority of patients scored above empirically identified screening cut-offs, despite the use of the most specific empirically-validated cut scores [10, 12]. Indeed, 43.4% of patients scored above the cut-off for likely internalizing disorder on the IDAS-D, and 20.1% of patients scored above the cut-off for likely Borderline Personality Disorder. Notably, patient reported mental health history much less frequently during clinical interview compared to self-report screening or symptom measures. However, even these lower rates greatly exceed population norms. These findings highlight both the frequency and severity of mental health concerns for individuals undergoing evaluation for EDS and hypermobility.

Whereas the majority of individuals with EDS or G-HSD reported mental health concerns, differences between diagnostic subgroups and those not diagnosed with EDS/hypermobility were less pronounced than in previous research [2]. In our sample, individuals diagnosed with G-HSD had greater odds of reporting a history of anxiety/depression than individuals with non-hypermobile EDS subtypes or those not diagnosed with EDS/hypermobility. There were no significant differences in rates of any other mental health diagnosis or on self-reported symptom severity measures. This is in contrast to the vast majority of prior reports, which identified higher rates of mental health disorders among individuals with EDS/G-HSD compared to matched controls [2, 7]. Indeed, even where there were significant differences (e.g. anxiety and depression) in this study, the effect size of these differences was much smaller than that reported in the past. There are several potential reasons why diagnostic groups in the present study did not differ in mental health concerns. It may be that such differences were not identified because data collection was not optimized for identification of mental health concerns. It may also be a reflection of cultural or systemic differences due to study of geographically restricted samples, both in this study and previous research. However, it is also possible that those not diagnosed with EDS/hypermobility in the present study represent a more well-matched comparison cohort that used in previous studies. These patients were all referred to the GoodHope EDS Clinic specifically for evaluation of possible EDS or hypermobility and in many cases this referral was due to presence of comorbidities common in EDS/G-HSD. Further research using control groups matched on multiple symptom measures (e.g. pain, autonomic dysfunction, dysphoria) are needed to isolate the relationship between collagen dysfunction or hypermobility and mental health outcomes.

The similarity in mental health concerns between individuals with EDS/G-HSD and those not diagnosed with EDS or G-HSD does not preclude the possibility of a unique mechanism underlying mental health concerns among individuals with EDS/G-HSD. However, if this is the case, the mechanism does not appear to be captured by the Beighton Score, which is the primary measure of hypermobility in the 2017 international diagnostic criteria [1, 17]. Future research should examine more nuanced relationships between connective tissue disorders and mental health symptoms. There are also several plausible shared mechanisms underlying the high levels of distress among patients assessed during this time period. In particular, other medical concerns, such as pain or autonomic dysfunction impacted individuals across diagnostic categories and are known to exacerbate psychological distress [10]. Indeed, these variables have been previously reported to be strongly associated with psychopathology among individuals with EDS/G-HSD [18, 19]. In the current study, these symptoms did not differ between individuals with hypermobility (hEDS, G-HSD) and those with chronic widespread pain and no hypermobility. In addition to these distressing medical symptoms, individuals with EDS/G-HSD often report a long history of invalidation from medical providers, friends, and family [20]. This invalidation can exacerbate symptoms such as pain [21], and has likely been experienced for many years, given that many individuals with EDS/G-HSD go 10 or more years from symptom onset to diagnosis. It is likely as well that those individuals who were referred to our clinic and did not receive a diagnosis of EDS or G-HSD have had many similar experiences. Future studies should focus on experimental and longitudinal evaluation of these putative mechanisms to identify novel and specific treatment targets for individuals with these complex medical concerns.

Given the prevalence of mental health concerns in this population, it is critical to provide mental health support to patients with EDS and G-HSD as a standard part of a multidisciplinary care approach. Research on efficacy of specific psychological interventions for EDS/G-HSD is limited, but there is a developing evidence base supporting cognitive-behavioral therapy as a self-management approach as well as third-wave behavior therapies (e.g. ACT, Acceptance and Commitment Therapy; DBT, Dialectic Behavior Therapy) [22]. At the GoodHope EDS Clinic, we provide a stepped-care treatment approach incorporating ACT and DBT in groups and individual psychotherapy focused on symptom management and adjustment to chronic illness [8]. However, it is important that providers take a patient-centred and trauma-informed approach to reduce stigma when referring patients with EDS or G-HSD to mental health services. Patient interest in such referrals may be limited due to perceptions that this implies symptoms are psychogenic [23]. 

There are several notable limitations to this study. First, these data do not include gold-standard mental health diagnoses. In the case of “likely” Borderline Personality Disorder in particular, we do not believe that high scores on this self-report measure represent true diagnosis, but instead is likely to capture emotion dysregulation associated with general distress, pain, and autonomic dysfunction. All mental health information was collected as part of a medical diagnostic evaluation with the goal of screening to facilitate referral to the GoodHope EDS Clinic psychology program [8]. The encounter in which the patient history taking occurred was a clinical encounter and not standardized for research. It is likely that there was substantial variation in how questions were asked and the exact timing of when measures were completed. It is also notable that there are potential biases in both self-report and clinician interview when assessing mental health as both are subject to recall bias and patients are potentially more likely to self-report than disclose to a clinician [23]. As data collection was not designed with this study in mind, only limited demographic information was collected. Moreover, the structure of these appointments changed after the beginning of the COVID-19 pandemic in March, 2020. In order to reduce face-to-face contact, the history component of the diagnostic appointment was separated from the physical exam and was conducted using secure telehealth programming. The physical exam then occurred at a later date. From a statistical perspective, many fewer patients were diagnosed with non-hypermobile EDS and hEDS than other diagnoses and statistical tests utilizing these categories should be interpreted with caution. Despite these limitations, this retrospective analysis included a large cohort of individuals with EDS/G-HSD and a well-matched comparison group. Future studies within the GoodHope EDS Clinic will focus on prospective evaluation of mental health and the impact of psychological intervention for individuals with EDS or G-HSD.

In conclusion, the vast majority of patients assessed at the GoodHope EDS Clinic reported mental health concerns in at least one domain. Indeed, rates of reported mental health diagnoses among all individuals assessed at the GoodHope EDS Clinic were well above the lifetime prevalence of these disorders in the general population. However, rates of mental health concerns largely did not differ between those diagnosed with EDS/G-HSD and those for whom EDS/G-HSD were ruled out but presented with a complex medical history including chronic pain. Future research should seek to determine if there are unique mechanisms underlying mental health concerns in EDS/G-HSD that could point to population specific treatment options.

## Data Availability

The data that support the findings of this study are not openly available due to reasons of sensitivity and are available from the corresponding author upon reasonable request. Data are stored in controlled access data storage at University Health Network.

## Abbreviations

BPD: Borderline personality disorder
BSL: Borderline symptom list
EDS: Ehlers-danlos syndrome
G-HSD: Generalize hypermobility spectrum disorder
hEDS: hypermobile ehlers-danlos syndrome
IDAS-D: Inventory of depressive and anxiety symptoms – dysphoria scale
MDHAQ: Multidimensional health assessment questionnaire
NP: Nurse practitioner
PTSD: Posttraumatic stress disorder
REB: Research ethics board
